# Unilateral Multicystic Dysplastic Kidney in an Infant: A Case Report of Surgical Nephrectomy, Diagnostic Imaging, and Long-Term Outcomes

**DOI:** 10.7759/cureus.92092

**Published:** 2025-09-11

**Authors:** Fatima Abeer, Ali Ahraz, Bisma Javid, Aisha Mahmood Ul Hassan, Gazala Andleeb

**Affiliations:** 1 Department of Medicine, Government Medical College, Srinagar, Srinagar, IND; 2 Department of Medicine, University of Dhaka, Dhaka, BGD; 3 Department of Internal Medicine, Government Medical College, Srinagar, Srinagar, IND; 4 Department of Pediatrics, Government Medical College, Srinagar, Srinagar, IND

**Keywords:** chronic kidney disease-mineral and bone disorder (ckd-mbd), compensatory renal hypertrophy, congenital solitary kidney, hyperfiltration injury, multicystic dysplastic kidney (mcdk), proteinuria in children, renal dysplasia

## Abstract

Multicystic dysplastic kidney (MCDK) is a common congenital renal anomaly often considered benign when unilateral and managed conservatively. We report a female infant diagnosed antenatally with left-sided MCDK in the setting of maternal gestational diabetes mellitus (GDM). Postnatal imaging confirmed a nonfunctioning left kidney and a structurally normal right kidney with reduced glomerular filtration rate (GFR) of 49.7 mL/min/1.73 m². Laparoscopic nephrectomy at six months revealed complete parenchymal replacement by cysts with focal cartilaginous metaplasia. Over four years of follow-up, the right kidney demonstrated progressive hypertrophy, yet biochemical surveillance showed persistently reduced GFR (52 mL/min/1.73 m²), proteinuria (0.32 g/L), hyperphosphatemia (4.68 mg/dL), and elevated alkaline phosphatase (197 U/L). This case highlights the discordance between structural compensation and functional sufficiency in solitary kidney physiology, the potential influence of prenatal metabolic factors on nephrogenesis, and the importance of proactive biochemical surveillance. Early detection of proteinuria and phosphate retention, combined with timely nephroprotective interventions, may help mitigate the long-term risk of chronic kidney disease in children with congenital solitary kidneys.

## Introduction

Multicystic dysplastic kidney (MCDK) is the most common congenital cystic renal disease, affecting approximately one in 4,300 live births [1]. It is characterized by the complete replacement of functional renal parenchyma with multiple noncommunicating cysts, loss of normal nephron architecture, and stromal fibrosis [2]. In most cases, the affected kidney is nonfunctional, a finding confirmed by radionuclide imaging. Historically, unilateral MCDK has been regarded as a benign condition with an excellent prognosis, leading to widespread adoption of conservative management with periodic ultrasonography and passive observation [3].

Emerging evidence challenges this benign view. Children with a congenital solitary kidney are at increased risk for hypertension, proteinuria, and progressive renal impairment, largely due to compensatory hyperfiltration injury in the contralateral kidney [4]. These changes may occur insidiously, and their onset can precede measurable declines in glomerular filtration rate (GFR). Early biochemical abnormalities, such as proteinuria, hyperphosphatemia, and elevated alkaline phosphatase signal, and subclinical renal stress before structural imaging detects damage [5]. This raises concern that reliance on ultrasonography alone may underestimate early functional compromise.

Histopathological findings may also refine risk assessment. The presence of cartilaginous metaplasia in MCDK reflects aberrant mesenchymal differentiation during nephrogenesis and may be associated with more severe developmental disruption, although its prognostic implications remain uncertain. Integrating such histological markers into follow-up strategies could improve patient stratification. In addition, prenatal factors may influence renal development. Maternal gestational diabetes mellitus (GDM) has been linked to impaired nephrogenesis through dysregulation of signaling pathways such as GDNF-RET and Pax2 [6]. Although this association is increasingly recognized, its impact on long-term renal outcomes in infants with MCDK is underexplored [7,8].

We present a case of a female infant with antenatally diagnosed unilateral MCDK who underwent laparoscopic nephrectomy and developed early biochemical evidence of renal impairment despite progressive hypertrophy of the contralateral kidney [9]. This case underscores the limitations of a purely structural surveillance strategy and supports the incorporation of biomarker-based monitoring in the management of children with congenital solitary kidneys [10,11].

## Case presentation

A female infant was born at 37 weeks of gestation in June 2019 via elective lower segment cesarean section at a tertiary care hospital in Srinagar, Jammu and Kashmir, India. The pregnancy was complicated by GDM diagnosed at 24 weeks and managed with insulin for the remainder of the pregnancy. Antenatal ultrasonography performed at 24 weeks revealed a left MCDK characterized by multiple noncommunicating cysts and absence of corticomedullary differentiation. The right kidney was structurally normal, and there were no signs of oligohydramnios or other congenital anomalies.

**Figure 1:**
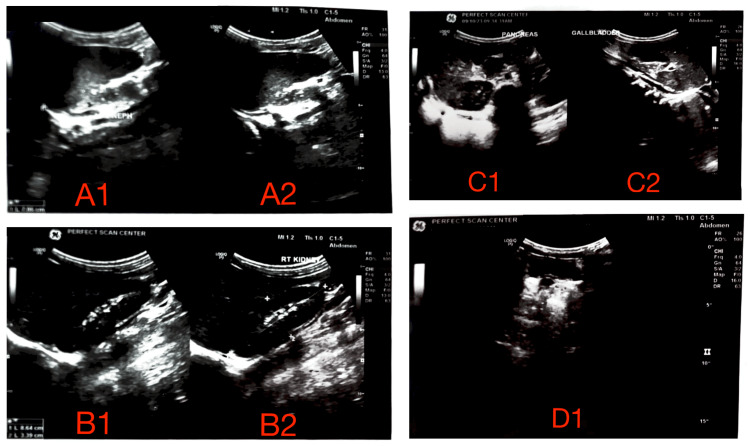
Preoperative ultrasonographic evaluation in the pediatric patient with unilateral MCDK. (A1) Longitudinal grayscale sonogram of the left kidney demonstrating complete replacement of renal parenchyma by multiple noncommunicating cysts of variable sizes, with total loss of corticomedullary differentiation, consistent with MCDK. (A2) Transverse grayscale view of the left kidney reaffirming diffuse cystic architecture without identifiable functional parenchyma. (B1) Longitudinal grayscale ultrasound of the contralateral right kidney revealing preserved corticomedullary differentiation, homogeneous echotexture, and renal length appropriate for age, without hydronephrosis, calculi, or focal lesions. (B2) Transverse grayscale view of the right kidney corroborating intact architecture and absence of structural abnormalities. (C1) Longitudinal abdominal scan visualizing the pancreas, showing normal size, homogeneous echotexture, and absence of focal masses or ductal dilatation. (C2) Longitudinal sonogram of the gallbladder displaying a thin, regular wall and anechoic lumen, without evidence of cholelithiasis or pericholecystic fluid. (D1) Color Doppler ultrasound of the right kidney demonstrating normal intrarenal vascular arborization with symmetric cortical perfusion and no perfusion defects, supporting adequate functional reserve of the solitary functioning kidney. MCDK, multicystic dysplastic kidney

Postnatal evaluation began at one month of age with a renal ultrasound that confirmed the antenatal findings. The left kidney measured 4.7 × 1.7 cm and was entirely cystic with no identifiable parenchyma. The right kidney measured 5.7 × 2.2 cm, with preserved corticomedullary differentiation and normal echogenicity (Figure 1). At two months, a technetium-99m dimercaptosuccinic acid (DMSA) scan showed no uptake by the left kidney and 100% cortical uptake by the right kidney, confirming functional absence of the left side. At three months, a technetium-99m diethylenetriaminepentaacetic acid (DTPA) renogram showed complete nonvisualization of the left kidney and estimated a GFR of 49.7 mL/min/1.73 m² for the right kidney - approximately 35% lower than the expected range for age. Delayed tracer washout (half-time of 1.5 minutes) was also noted, suggesting subclinical tubular dysfunction (Table 1).

**Table 1:** Patient demographics, antenatal findings, and perioperative vitals PR, pulse rate; RR, respiratory rate; HR, heart rate; MCDK, multicystic dysplastic kidney

Parameter	Value
Sex	Female
Age at renal scintigraphy	2 mo (Sep 5, 2019)
Age at pre‑op evaluation	5 mo (Oct 10, 2019)
Age at surgery/discharge	7 mo (Jan 10 - Jan 14, 2020)
Weight (at 5 mo)	8.0 kg
Primary diagnosis	Unilateral left MCDK
Antenatal finding	Oligohydramnios, labeled “polycystic disease” on prenatal scan
Preoperative HR	160 bpm
Admission vitals (day 0)	PR 125/min, RR 33/min

Given the risk of mass effect, anatomical distortion, and future infection, a laparoscopic left nephrectomy was performed at six months of age. Intraoperative findings included complete replacement of renal parenchyma with multiloculated cysts ranging from 0.1 to 4.4 cm in diameter. The ureter was atretic, measuring approximately 2 cm in length, and the kidney was adherent to surrounding omental tissue, requiring careful dissection. The surgery was completed uneventfully, and the patient was discharged on postoperative day four with a course of oral cefuroxime. Serial laboratory investigations before and after surgery are summarized in Table 2.

**Table 2:** Serial laboratory investigations before and after surgery Values represent serial laboratory test results obtained preoperatively and at various postoperative time points. Units are reported in SI format where applicable. Reference ranges correspond to institutional standards at the time of testing. ALT, alanine aminotransferase; ALP, alkaline phosphatase; WBC, white blood cell count; HPF, high-power field; SI, International System of Units

Date	Parameter	Result	Unit	Reference Range
Pre-op	Hemoglobin	9.9	g/dL	10.5-13.5
Pre-op	Glucose (random)	190	mg/dL	70-140
Pre-op	Sodium	8.1	mmol/L	135-145
Pre-op	Urea	190	mg/dL	10-50
16-Sep-2019	WBC	6.3	×10⁹/L	5-19.5
16-Sep-2019	Neutrophils	70	%	30-60
16-Sep-2019	Platelets	277	×10⁹/L	150-450
16-Sep-2019	Hemoglobin	8.9	g/dL	10.5-13.5
16-Sep-2019	Bicarbonate	20.9	mmol/L	22-28
16-Sep-2019	Calcium	10.2	mg/dL	8.5-10.5
16-Sep-2019	Sodium	135	mmol/L	135-145
16-Sep-2019	Potassium	4.5	mmol/L	3.5-5.0
16-Sep-2019	Bilirubin (total)	0.68	mg/dL	<1.2
16-Sep-2019	ALT	49	U/L	<45
16-Sep-2019	ALP	422	U/L	40-130
16-Sep-2019	Albumin	4	g/dL	3.9-4.9
14-Jan-2020	Urea	0.7	mmol/L	1.2-5.7
14-Jan-2020	Creatinine	60	µmol/L	<70
14-Jan-2020	ALT	210	U/L	<45
14-Jan-2020	ALP	342	U/L	40-130
14-Jan-2020	Albumin	9.9	g/dL	3.9-4.9
25-Jul-2022	Urine colour	Yellowish	-	Normal
25-Jul-2022	Urine pH	Acidic	-	<6
25-Jul-2022	Leucocytes	1-2	/HPF	0
25-Jul-2022	Epithelial cells	Positive	-	Negative
25-Jul-2022	Bacterial flora	Positive	-	None detected
25-Jul-2022	Urine glucose	Negative	%	0.5-1
25-Jul-2022	Urine protein	Negative	-	Negative

Histopathological examination confirmed multicystic renal dysplasia. The specimen revealed numerous cysts lined by cuboidal to flattened epithelium embedded in dense fibrous stroma, along with primitive tubular elements and rudimentary glomeruli. Notably, multiple foci of mature hyaline cartilage were identified, indicating aberrant mesenchymal differentiation during nephrogenesis. No normal renal parenchyma was present in any section examined.

**Figure 2:**
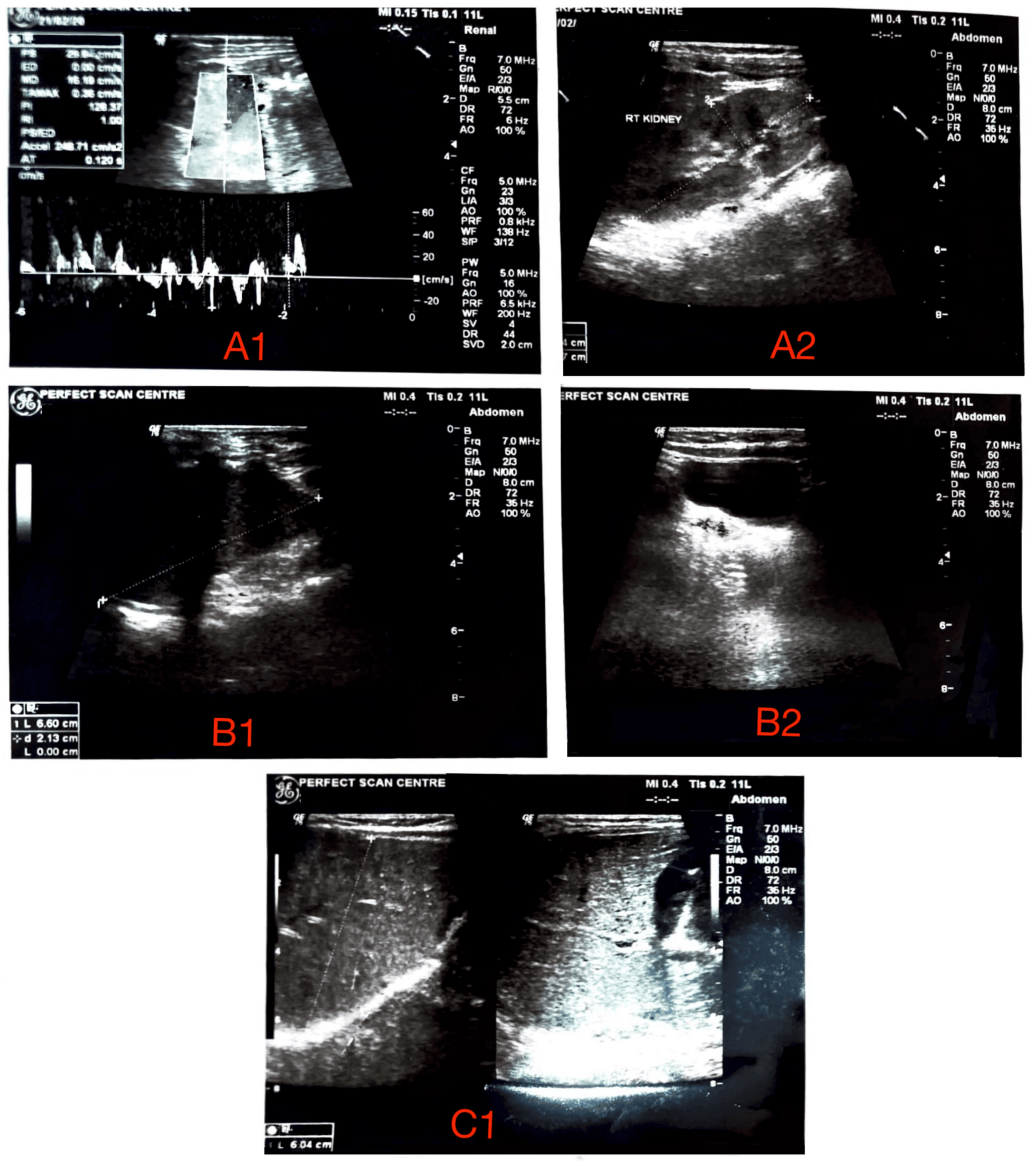
Postoperative Doppler and grayscale ultrasonographic assessment in the pediatric patient with a history of unilateral MCDK status post left nephrectomy. (A1) Renal artery spectral Doppler waveform of the right kidney demonstrating normal resistive index and acceleration time, indicating preserved vascular patency and hemodynamics. (A2) Longitudinal grayscale sonogram of the right kidney showing intact corticomedullary differentiation, homogeneous parenchymal echotexture, and normal dimensions for age, with no evidence of hydronephrosis or focal lesions. (B1) Longitudinal grayscale view of the right kidney with caliper measurements confirming appropriate renal length and cortical thickness. (B2) Transverse grayscale view of the right kidney reaffirming normal renal architecture without cystic or solid masses. (C1) Longitudinal and transverse grayscale views of the right kidney hilum displaying normal renal sinus echogenicity, intact perihilar structures, and no perinephric fluid collections, consistent with a healthy, solitary functioning kidney. MCDK, multicystic dysplastic kidney

The postoperative recovery was uncomplicated, with appropriate wound healing and no signs of infection or adverse events. Serial ultrasound monitoring over the following four years demonstrated progressive compensatory hypertrophy of the right kidney, which increased in size from 6.2 × 2.4 cm at six months to 8.6 × 3.3 cm by four years of age. The right kidney remained structurally normal without hydronephrosis, nephrolithiasis, or scarring (Figure 2).

Despite adequate structural compensation, biochemical surveillance revealed early evidence of renal impairment. At four years of age, serum creatinine was 0.4 mg/dL and estimated GFR by the Schwartz formula was 52 mL/min/1.73 m². Urinalysis revealed proteinuria (0.32 g/L), confirmed through 24-hour urine collection, along with creatinine excretion of 216.73 mg/dL. Serum phosphorus was elevated at 4.68 mg/dL (normal range 3.8-4.5 mg/dL), and alkaline phosphatase was also elevated at 197 U/L. Parathyroid hormone and calcium levels remained within normal limits. Throughout the follow-up period, the patient’s blood pressure remained normal, with the most recent measurement being 94/61 mmHg, corresponding to the 50th percentile for age and height.

The patient continues to grow and develop normally, with height and weight tracking at the 75th percentile. Current management focuses on renal preservation and includes calcium carbonate (250 mg daily), cholecalciferol (400 IU daily), dietary phosphorus restriction, and moderate protein limitation. A structured surveillance protocol has been implemented, consisting of quarterly monitoring of blood pressure, urinalysis, and urine protein-to-creatinine ratio, semi-annual serum biochemistry, including phosphorus and alkaline phosphatase, and annual renal ultrasound. Repeat nuclear medicine imaging is planned if there is any clinical deterioration. The patient remains under close nephrological follow-up to monitor progression and prevent long-term complications such as chronic kidney disease.

## Discussion

This case illustrates the limitations of relying solely on anatomical adaptation when assessing outcomes in children with unilateral MCDK. Although the patient’s contralateral kidney demonstrated progressive hypertrophy over four years, GFR remained persistently reduced, and early biochemical abnormalities, including proteinuria and hyperphosphatemia, were detected. These findings emphasize that structural compensation does not always equate to functional sufficiency in solitary kidney physiology and that early renal stress can occur in the absence of overt symptoms.

Traditionally, unilateral MCDK with a normal contralateral kidney has been considered a benign anomaly, leading to conservative management strategies focused on periodic ultrasonography and blood pressure measurement. However, longitudinal studies suggest that 30-40% of children with a congenital solitary kidney develop reduced renal function, proteinuria, or hypertension in adolescence or early adulthood [2]. Hyperfiltration injury is thought to be a key driver of this decline, with sustained increases in single-nephron filtration pressure eventually leading to glomerulosclerosis. In this patient, the presence of subnephrotic proteinuria and reduced GFR from infancy supports the likelihood of ongoing hyperfiltration-related stress despite normal clinical status.

The biochemical profile in this case is notable for isolated hyperphosphatemia and elevated alkaline phosphatase in the setting of normal calcium and parathyroid hormone levels. These abnormalities may indicate early proximal tubular dysfunction or altered phosphate handling, possibly mediated by downregulation of sodium-phosphate cotransporters, such as NaPi-IIa, in the context of hyperfiltration. Although serum phosphate is not routinely monitored in pediatric solitary kidney follow-up, emerging evidence suggests it may be an under-recognized marker of subclinical nephron stress. Early detection of such abnormalities provides an opportunity for timely intervention before irreversible damage occurs.

Histopathological analysis revealed multiple foci of mature hyaline cartilage within the dysplastic kidney, consistent with cartilaginous metaplasia. This represents a severe form of developmental disruption arising from aberrant mesenchymal differentiation during nephrogenesis. While the prognostic significance of this finding remains unclear, some reports suggest it may correlate with a lower likelihood of spontaneous involution and a higher risk of complications. Integrating histopathological features into prognostic models could enhance risk stratification and inform the intensity of follow-up.

An additional consideration in this case is the maternal history of GDM, which has been associated with impaired nephrogenesis in experimental and epidemiological studies. Hyperglycemia during pregnancy can disrupt signaling pathways critical to kidney development, including GDNF-RET and Pax2, leading to reduced nephron number and abnormal branching morphogenesis. Although a causal relationship cannot be established here, the combination of severe structural derangement and abnormal histologic differentiation raises the possibility that prenatal metabolic factors contributed to disease severity. Given the global prevalence of gestational diabetes, further investigation into its role in congenital renal anomalies is warranted.

The clinical implications of this case support a shift from passive observation toward proactive, biomarker-driven surveillance in children with congenital solitary kidneys. While ultrasonography remains important for structural monitoring, it should be supplemented by regular assessment of urine protein-to-creatinine ratio, serum phosphate, and GFR. In patients with proteinuria, early initiation of angiotensin-converting enzyme inhibitors may reduce intraglomerular pressure, mitigate hyperfiltration injury, and slow progression toward chronic kidney disease, even in the absence of hypertension. Long-term nephrological follow-up into adolescence and adulthood is essential, as functional decline can occur gradually and remain clinically silent until advanced.

In summary, this case challenges the assumption that unilateral MCDK with a normal contralateral kidney is inherently benign. The persistence of biochemical abnormalities despite structural compensation underscores the importance of integrating functional and biochemical parameters into follow-up protocols. Recognizing early markers of renal stress offers an opportunity for intervention that may preserve long-term kidney function and improve outcomes in this vulnerable population.

## Conclusions

This case demonstrates that unilateral MCDK, even with a structurally normal contralateral kidney and evidence of compensatory hypertrophy, can be accompanied by early functional decline detectable through biochemical surveillance. Persistent reduction in GFR, proteinuria, and hyperphosphatemia in this patient highlights the inadequacy of relying solely on anatomical monitoring. The histological finding of cartilaginous metaplasia suggests a severe developmental disturbance, and the maternal history of gestational diabetes raises the possibility of prenatal metabolic influence on renal morphogenesis. These observations support a shift toward proactive, individualized follow-up that integrates regular monitoring of proteinuria, serum phosphate, and renal function from infancy, with early nephroprotective therapy such as angiotensin-converting enzyme inhibitors when indicated. Lifelong nephrological surveillance throughout adolescence and adulthood is essential for detecting and mitigating progression toward chronic kidney disease. Further studies are needed to clarify the prognostic value of histological features and maternal risk factors in this setting.

## References

[1] Meyers ML, Treece AL, Brown BP, Vemulakonda VM (2020). Imaging of fetal cystic kidney disease: multicystic dysplastic kidney versus renal cystic dysplasia. Pediatr Radiol.

[2] Kaissling B, Le Hir M (2008). The renal cortical interstitium: morphological and functional aspects. Histochem Cell Biol.

[3] Cardona-Grau D, Kogan BA (2015). Update on multicystic dysplastic kidney. Curr Urol Rep.

[4] G Kalaitzidis R (2021). Should we need more sensitive early diagnostic markers in children with congenital solitary functioning kidneys?. J Clin Hypertens.

[5] Grenier N, Merville P, Combe C (2016). Radiologic imaging of the renal parenchyma structure and function. Nat Rev Nephrol.

[6] Tortelote GG (2024). The impact of gestational diabetes on kidney development: is there an epigenetic link?. Curr Diab Rep.

[7] Baker HM, Jnah AJ (2024). Supporting infants with multicystic dysplastic kidney disease: a comprehensive approach. Neonatal Netw.

[8] Rubio-Aliaga I, Krapf R (2022). Phosphate intake, hyperphosphatemia, and kidney function. Pflugers Arch.

[9] Alexander RT, Dimke H (2023). Effects of parathyroid hormone on renal tubular calcium and phosphate handling. Acta Physiol (Oxf).

[10] Cozzolino M, Ketteler M, Wagner CA (2020). An expert update on novel therapeutic targets for hyperphosphatemia in chronic kidney disease: preclinical and clinical innovations. Expert Opin Ther Targets.

[11] Ataga KI, Saraf SL, Derebail VK (2022). The nephropathy of sickle cell trait and sickle cell disease. Nat Rev Nephrol.

